# Plasma cell neoplasia after kidney transplantation: French cohort series and review of the literature

**DOI:** 10.1371/journal.pone.0179406

**Published:** 2017-06-21

**Authors:** Raphaël Kormann, Hélène François, Thibault Moles, Jacques Dantal, Nassim Kamar, Karine Moreau, Thomas Bachelet, Anne-Elisabeth Heng, Antoine Garstka, Charlotte Colosio, Didier Ducloux, Johnny Sayegh, Benjamin Savenkoff, Denis Viglietti, Rebecca Sberro, Eric Rondeau, Julie Peltier

**Affiliations:** 1 Service d'Urgences Néphrologiques et Transplantation Rénale, Hôpital Tenon, APHP, Université Pierre et Marie Curie, Paris, France; 2 Service de Néphrologie, Hôpital Bicêtre, APHP, Université Paris Sud, Paris, France; 3 Service de Néphrologie et d'Immunologie Clinique, Centre Hospitalier Universitaire de Tours, Tours, France; 4 Service de Néphrologie et d'Immunologie Clinique, Centre Hospitalier Universitaire de Nantes, Nantes, France; 5 Service de Néphrologie et Transplantation, CHU Rangueil, Toulouse, Toulouse, France; 6 Service de Néphrologie et Transplantation, Centre Hospitalier Universitaire de Bordeaux, Bordeaux, France; 7 Centre de Traitement des Maladies Rénales-Clinique Saint Augustin, 96 avenue d’Arès, Bordeaux, France; 8 Service de Néphrologie—Hémodialyses, Centre Hospitalier Universitaire Gabriel-Montpied, Clermont-Ferrand, France; 9 Service de Néphrologie, Centre Hospitalier Regional Universitaire de Lille, Lille, France; 10 Service de Néphrologie—Hypertension artérielle—Hémodialyse—Transplantation, Centre Hospitalier Universitaire de Reims, Reims, France; 11 Service de Néphrologie-Dialyse, Centre Hospitalier Régional Universitaire, Hôpital Jean Minjoz, Besançon, France; 12 Service de Néphrologie—Dialyse—Transplantation, Centre Hospitalier Universitaire d'Angers, Angers, France; 13 Service de Néphrologie et Transplantation, Centre Hospitalier Universitaire de Nancy, Vandoeuvre-Les-Nancy, France; 14 Service de Néphrologie, Hôpital Saint Louis, Université Denis Diderot-Paris VII AP-HP, Paris, France; 15 Service de Transplantation, Hôpital Necker, Université Paris Descartes AP-HP, Paris, France; Johns Hopkins School of Medicine, UNITED STATES

## Abstract

Although post-transplant lymphoproliferative disorder (PTLD) is the second most common type of cancer in kidney transplantation (KT), plasma cell neoplasia (PCN) occurs only rarely after KT, and little is known about its characteristics and evolution. We included twenty-two cases of post-transplant PCN occurring between 1991 and 2013. These included 12 symptomatic multiple myeloma, eight indolent myeloma and two plasmacytomas. The median age at diagnosis was 56.5 years and the median onset after transplantation was 66.7 months (2–252). Four of the eight indolent myelomas evolved into symptomatic myeloma after a median time of 33 months (6–72). PCN-related kidney graft dysfunction was observed in nine patients, including six cast nephropathies, two light chain deposition disease and one amyloidosis. Serum creatinine was higher at the time of PCN diagnosis than before, increasing from 135.7 (±71.6) to 195.9 (±123.7) μmol/l (p = 0.008). Following transplantation, the annual rate of bacterial infections was significantly higher after the diagnosis of PCN, increasing from 0.16 (±0.37) to 1.09 (±1.30) (p = 0.0005). No difference was found regarding viral infections before and after PCN. Acute rejection risk was decreased after the diagnosis of PCN (36% before versus 0% after, p = 0.004), suggesting a decreased allogeneic response. Thirteen patients (59%) died, including twelve directly related to the hematologic disease. Median graft and patient survival was 31.7 and 49.4 months, respectively. PCN after KT occurs in younger patients compared to the general population, shares the same clinical characteristics, but is associated with frequent bacterial infections and relapses of the hematologic disease that severely impact the survival of grafts and patients.

## Introduction

Risk of neoplasia is increased in solid organ transplantation [1], and post-transplant lymphoproliferative disorder (PTLD) is the second most common type of cancer, after skin cancer [2]. According to the International Society of Hematology, plasma cell neoplasia (PCN), including multiple myeloma (MM) and plasmacytoma occurring in solid organ transplantation, forms part of monoclonal PTLD.

MM is a disorder of post-germinative B-cell malignant proliferation, in which a monoclonal immunoglobulin (M-protein) is secreted. MM is consistently preceded by a pre-tumoral stage known as monoclonal gammopathy of undetermined significance (MGUS) [3,4]. After several chromosomal and genetic events, MGUS can move towards smoldering multiple myeloma (SMM), MM and eventually plasma cell leukemia [5,6].

Actual knowledge about PCN in kidney transplantation (KT) is scarce. PCN arising after KT represents only 4% of PTLD in KT in France [7]. An epidemiologic study in the United States performed in 202,600 solid organ transplant recipients between 1987 and 2009 estimated a prevalence of 0.7/1000 [8]. Although PCN occurs very rarely in KT, relative risk compared with the general population is increased two-fold in KT, and is significantly higher for plasmacytoma than for MM [8]. Relative risk is also higher in patients younger than 35 years, and Epstein-Barr virus (EBV) seronegative patients [8].

Until recently, only case reports of PCN arising after KT have been reported in the literature. Kidney graft plasmacytomas have been reported in five patients [9–13]. The tumor originated from the donor in three cases [9,10,12]. Four multiple plasmacytomas [14–16] and nine isolated plasmacytomas [16–24] located outside the graft have been described. Among 11 patients tested, EBV in situ hybridization was positive in seven cases [14–16,18,19, 21,24], suggesting the virus played a role in disease development. Thirteen patients developing MM after KT have been reported in case reports from 1983 to 2011, but no conclusion could be made about their particularities and prognosis [25–28]. Recently, Safadi et al. [29] reported seven cases of MM occurring after KT between 2001 and 2012. Most of these patients had MGUS before KT, and MM could occur at any time after transplantation. Graft failure was often related to the evolution of the hematologic disease. Median survival was 80 months [29], and comparable to the general population [30].

In the present study, we report 22 cases of PCN emerging after KT over a period of 22 years (1991 to 2013) in France.

## Materials and methods

### Patients

This was a multicenter, retrospective, cohort study conducted at 14 French renal transplantation centers. A questionnaire was sent to the 33 French centers involved in renal transplantation in order to recruit renal transplanted patients with PCN. Fourteen centers responded favorably and 22 patients were included after identification through the computerized records. Medical records of patients were accessed and data were analyzed anonymously. Inclusion criterion was PCN (SMM, MM or solitary plasmacytoma) occurring after renal transplantation. MGUS non-evolving to MM was not included. The diagnosis of SMM, MM and plasmacytoma was based on the criteria of the International Myeloma Working Group [31]. Responses to treatments were classified according to the International Myeloma Working Group criteria [32].

Related-organ or tissue impairments (ROTI) were as follows: hypercalcemia defined by serum calcium > 0.25 mmol/L (>1 mg/dL) higher than the upper limit of normal or >2.75 mmol/L (>11 mg/dL), renal insufficiency defined by creatinine clearance <40 ml/min or serum creatinine >177 μmol/L (>2 mg/dL), anemia defined by hemoglobin value of >20 g/L below the lower limit of normal, or a hemoglobin value <100 g/L, presence of one or more osteolytic bone lesions [31].

Demographic, clinical and laboratory data were assessed for each patient before kidney transplantation (sex, previous history of non-hematologic or hematologic neoplasia, age at End Stage Renal Disease (ESRD), native kidney disease, dialysis duration, number of previous KT); at the time of renal transplantation (age at KT, type of induction therapy, initial maintenance immunosuppression); and after KT, before and after the diagnosis of PCN (serum creatinine evolution, acute or chronic graft rejection, infections (bacterial, viral and fungal), evolution of immunosuppressive treatments, transplant status, dialysis post-transplant, and graft loss and patient outcome (death)). Data about PCNs were also precisely recorded (age at diagnosis, time of diagnosis, time after KT, known monoclonal peak before KT, known monoclonal peak after KT, paraprotein at MM diagnosis, medullary plasmacytosis at diagnosis, M Spike level at diagnosis, progression of SMM to MM, Bence Jones proteinuria, type of ROTI, new types of ROTI during follow up, related plasma-cell related disorders (plasmacytomas, hyperviscosity, amyloïdosis), treatments, and relapses). Progression free survival was defined as the time from the first dose of the first treatment for PCN to disease progression or death, whichever came first.

### Statistical analysis

Quantitative data are presented as mean (1 standard deviation) or median (range).

The prevalence rate was calculated from the number of cases of PCN occurring between 2002 and 2013 (19 cases), and the number of patients aged over 18 receiving KT in the 14 transplantation centers during the same period. The French Biomedical Agency (ABM) provided the exact number of KTs during this period in these centers (14 551 KTs). As data before 2002 was not accurately recorded, three older cases of PCN were excluded from the calculation.

The annual rate of bacterial and viral infection was compared before and after the diagnosis of PCN, and each patient was his own control.

Serum creatinine was compared before and at the time of diagnosis of PCN. Creatinine before diagnosis was either nadir creatinine for patients with early onset of PCN <1 year after KT, or creatinine measured 1 year ± 2 months before diagnosis of PCN for patients with later PCN, > 1 year after KT.

The Wilcoxon matched-pairs signed rank test was used to compare the annual bacterial or viral infection rate before and after the diagnosis of PCN, and serum creatinine before and at the time of diagnosis of PCN.

The Mann Whitney test was used to compare the onset of PCN of patients with or without a known MGUS before KT.

The number of acute rejections before and after the diagnosis of PCN for each patient was compared using Fisher’s exact test.

Analysis of patient and graft survival uses Kaplan-Meier curves.

All comparisons are two sided and a value of p<0.05 is considered significant.

## Results

### Characteristics of patients and renal transplantation

Twenty-two patients (15 men and seven women, odds ratio = 2.1), who developed PCN after KT were included. The patients were transplanted between 1986 and 2012 and PCN was diagnosed after KT between 1991 and 2013. Characteristics of patients and KT are presented in **Table 1.**

**Table 1:** Characteristics of patients and kidney transplantation.

Patient	Initial diagnosis of the PCN	Sex	Native kidney disease	Dialysis duration (year)	Number of previous KT	Age at KT	Induction therapy	Initial Maintenance immunosuppression	Acute rejection episodes	chronic graft dysfunction and chronic rejection	Transplant status	Dialysis post transplant	Outcome
1	MM	Male	diabetic nephropathy	3.6	1	50.4	ATG	Ciclosporine Imurel Prednisone	0		Failed allograft to cast nephropathy	yes	Dead (disseminated Aspergillosis)
2	MM	Female	toxic chronic tubulo-interstitial nephropathy	?		58.6	ATG	Ciclosporine Imurel	0		Dead with a functionnal graft	no	Dead (hemorragic stroke)
3	MM	Male	unknown	13.7		46.8	ATG	Ciclosporine Cellcept Prednisone	1	chronic graft dysfunction	Failed allograft to chronic graft dysfunction	yes	Alive in hemodialysis
4	MM	Female	Congenital dysplasia	0.9		35.9	ATG	Ciclosporine Imurel	1	chronic rejection	Failed allograft to LCDD	yes	Dead (disease progression)
5	MM	Male	Autosomic Dominant Polycystic Kidney Disease	1		48.5	?	?	1		Dead with a functionnal graft	no	Dead (disease progression)
6	MM	Male	Chronic glomerulopathy	1.5		61.8	Basiliximab	Tacrolimus Cellcept Prednisone	0		Failed allograft to cast nephropathy	yes	Dead (pneumoniae)
7	MM	Female	chronic tubulo-interstitial nephropathy	3		65.5	Basiliximab	Tacrolimus Cellcept Prednisone	0		Dead with a functionnal graft	no	Dead (disease progression)
8	MM	Male	unknown	5.4		68.9	ATG	Ciclosporine Cellcept Prednisone	0		Dead with a functionnal graft	no	Dead (disease progression)
9	MM	Male	Autosomic Dominant Polycystic Kidney Disease	9.1		65.1	Basiliximab	Tacrolimus Cellcept Prednisone	0		cast nephropathy	no	DEAD
10	MM	Male	hypertensive nephropathy	0.7		56.5	Basiliximab	Tacrolimus Cellcept Prednisone	0		cast nephropathy	no	Alive
11	MM	Male	unknown	?		27.2	?	Ciclosporine Cellcept Prednisone	0		functionnal graft	no	Alive
12	MM	Female	unknown	7.2		65.2	Basiliximab	Sirolimus Cellcept Prednisone	0		cast nephropathy	no	Alive
13	SMM	Male	Alport syndrome	23.6		43.2	ATG	Ciclosporine Cellcept Prednisone	0		cast nephropathy. Dead with a functionnal graft	no	Dead (pneumoniae)
14	SMM	Female	Membranous glomerulopathy	7.8		43.3	ATG	Ciclosporine Cellcept Prednisone	1		Dead with a functionnal graft	no	Dead (disease progression)
15	SMM	Male	Chronic glomerulopathy	6		28.6	ATG	Ciclosporine Imurel Prednisone	1		Dead with a functionnal graft	no	Dead (disease progression)
16	SMM	Male	unknown	0.9		43.9	ATG	Ciclosporine Cellcept Prednisone	0		Failed allograft to LCDD	yes	Dead (sepsis)
17	SMM	Male	AA Amyloidosis	15		71.5	Basiliximab	Tacrolimus Cellcept Prednisone	1		functionnal graft	no	Alive
18	SMM	Male	Kimura's disease	13.3	1	41	ATG	Tacrolimus Cellcept Prednisone	1	chronic rejection	Failed allograft to chronic rejection	yes	Alive
19	SMM	Male	membranoproliferative glomerulopathy	1.5	1	46.7	ATG	?	0	chronic rejection	functionnal graft	no	Alive
20	SMM + Amyloidosis	Female	chronic tubulo-interstitial nephropathy	?	2	47	ATG	Tacrolimus Cellcept Prednisone	0		Renal Amyloidosis. Dead with a functionnal graft	no	Dead (cardiac failure)
21	Plasmacytoma	Male	Chronic glomerulopathy	2.3		48	ATG	Ciclosporine Cellcept Prednisone	1		functionnal graft	no	Alive
22	Plasmacytoma	Female	vesicoureteral reflux	?		35.2	?	?	0		Dead with a functionnal graft	no	Dead (Disease progression)

MM: Multiple myeloma. SMM: Smoldering myeloma. KT: kidney transplantation. ATG: anti-thymocyte globulin.

The calculated prevalence rate was 1.3/1000. The median age at end stage renal disease was 46.2 years (19.2–63.5; 18/22 patients). Three patients had a previous history of non-hematologic neoplasia, and one had a previous history of benign B-cell proliferation. The median age at renal transplantation was 47.5 years (27.2–71.5). Two patients were transplanted from a living donor. One patient had a dual kidney transplant, and one a combined kidney-pancreas transplant.

### Characteristics of PCN after renal transplantation

#### General characteristics

**Fig 1:**
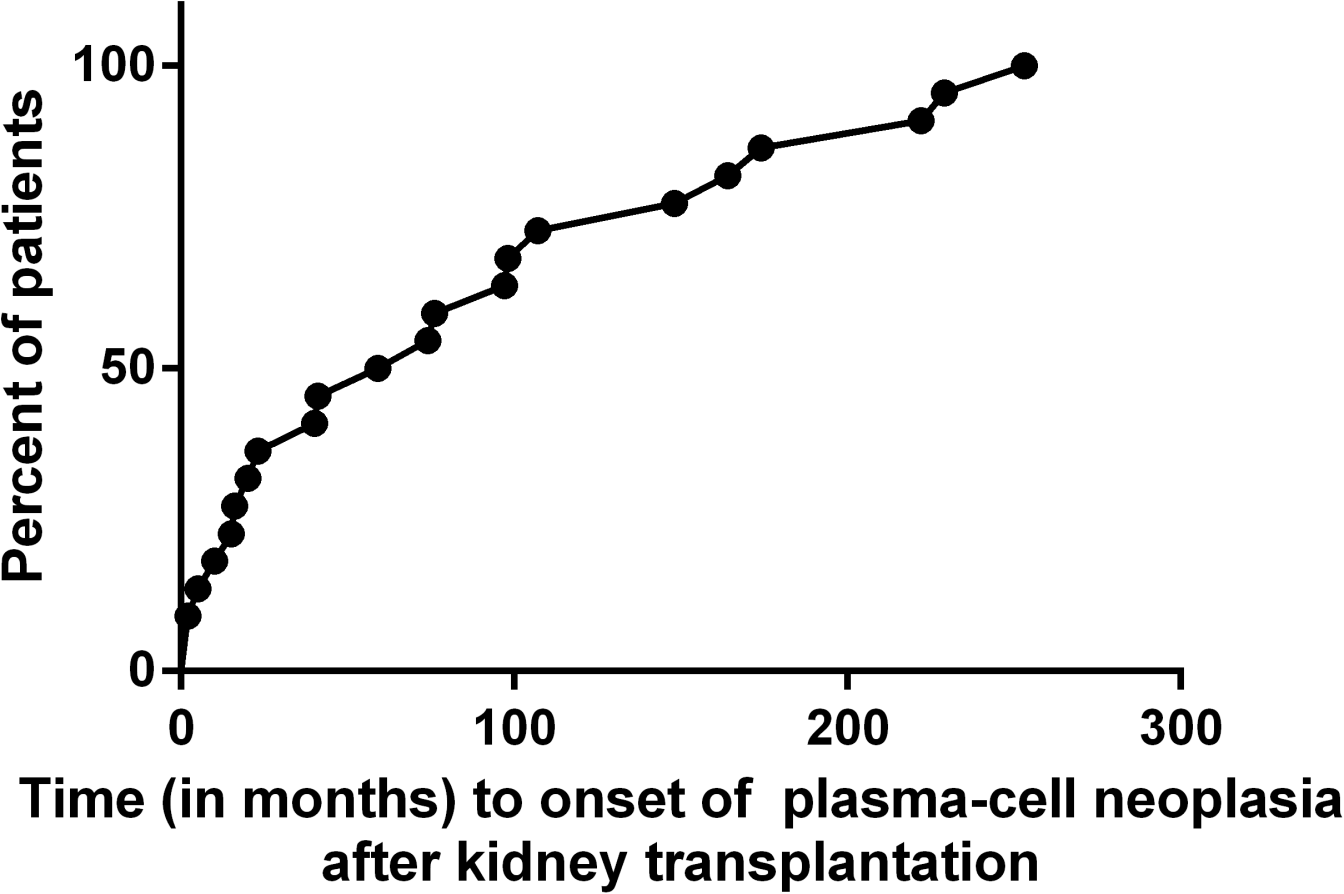
Time to onset of plasma cell neoplasia after kidney transplantation.

The median age at diagnosis of PCN was 56.5 years (41.8–72.3). At diagnosis, 12 patients had MM, eight with SMM and two with solitary plasmacytoma. The occurrence of PCN appeared to be constant over time. It could be diagnosed at any time after KT, from 2 to 252 month(s), with a median time of 66.7 months **(Fig 1).** Only eight patients had known MGUS before renal transplantation (36%), but they did not develop PCN significantly earlier after KT compared to patients without known monoclonal peak (respectively 53.5±67.9 versus 103.4 ±83.4 months, p = 0.18). Moreover, one patient had IgA lambda MGUS progressing as a lambda light chain myeloma, and another patient had IgA kappa MGUS and developed IgG kappa MM, suggesting either immunoglobulin class switch recombination, or the development of a new clonal plasma cell. Among the fourteen patients with no MGUS detected before KT (64%), five had light chain monoclonal proteins.

Of the 12 MM patients, six had kidney graft failure: five had cast nephropathy, three of which were confirmed by biopsy, and one had biopsy-confirmed light chain deposition disease (LCDD). The two remaining patients were considered as having cast nephropathy because of the association of acute kidney failure and massive proteinuria, composed of large amounts of monoclonal free light chains.

Four out of the eight SMMs progressed to MM during follow-up. One patient had cast nephropathy-related kidney graft failure and one evolved to plasma cell leukemia. Progression from SMM to MM was a median of 33 months (6–72). In the four non-progressing patients, one had amyloid light chain (AL) amyloidosis related to a kappa light chain, with cardiac and kidney graft failure. One had biopsy-confirmed graft failure due to LCDD, which was related to a kappa light chain. One had hyperviscosity syndrome, with an IgA lambda protein, but no end-organ damage. SMM persisted in the last patient at the end of follow-up.

Of the two patients with plasmacytoma at diagnosis, one patient had two solitary plasmacytomas in the sternum and the sacrum, which relapsed with multiple localizations (bone, gut and brain) after treatment. The other had a solitary plasmacytoma in the thoracic spine, which relapsed as multiple plasmacytomas. There was no bone marrow involvement in either patient.

The general characteristics of patients are summarized in **Table 2**.

**Table 2:** Characteristics of plasma-cell neoplasias after kidney transplantation.

Patient	Initial diagnosis of the PCN	Age at PCN diagnosis	Time of diagnosis	Time after KT (in month)	Paraprotein prior to KT	Paraprotein after KT	Paraprotein at MM diagnosis	Medullary plasmacytosis at diagnosis	M Spike (g/L)	Free light chain (mg/l)	Bence jones proteinuria	Related Organ or Tissu Impairment (ROTI) at diagnosis	New type of ROTI during follow up	Related plasma-cell related disorders
1	MM	51.6	1991	15	no	no	IgG kappa	13%	33		yes	Hypercalcemia, Bone, renal insuffisiency		
2	MM	67.6	1997	107	no	IgG lambda	IgG lambda	*	35		*	Bone, with medullar compression		
3	MM	53	2005	74	no	no	IgG kappa	13%	20.6		*	Bone, anemia		
4	MM	55	2006	229	no	IgG kappa	IgG kappa	51%	59		yes	Bone, anemia, renal insuffisiency		
5	MM	56.8	2006	98	no	no	kappa	*		*	*	Bone		
6	MM	62.2	2006	5	IgA kappa	* *	IgG kappa	70%	54		yes	Hypercalcemia, Bone, renal insuffisiency		
7	MM	66.8	2010	23	IgG kappa		IgG kappa	20%	10		no	Bone		
8	MM	72.3	2010	16	IgG lambda		IgG lamda	14%	9.7		no	Bone, anemia		
9	MM	68.5	2012	40	no	no	lambda	32% + haemophagocytic syndrome		1080	yes	Anemia, renal insuffisiency		
10	MM	57.4	2013	41	IgA lambda		lambda	53%		5200	yes	Hypercalcemia, Bone, renal insuffisiency		
11	MM	41.8	2013	10	no	IgG lambda	IgG lambda	Bone marrow and plasmacytoma biopsies	59.6		no	Bone		Huge iliac plasmacytoma
12	MM	66.9	2013	174	IgG lambda		IgG lambda	20%	14		yes	Renal insuffisiency		
13	SMM	43.3	1996	20	IgG lambda		IgG lambda	13%	30		no		Bone, anemia, renal insuffisiency	
14	SMM	48.2	2004	2	IgG kappa		IgG kappa	15%	14.7		no		Plasma cell leukemia and anemia	
15	SMM	47	2009	59	no	no	IgG kappa	10%	*		no		Bone, anemia	Frontal plasmacytoma
16	SMM +LCDD	45.9	2010	222	no	no	kappa	16%		964	yes	Renal insuffisiency		
17	SMM	71.5	2010	2	no	no	kappa	11%		1000	yes		Bone	
18	SMM	49	2011	97	no	IgA lambda	IgA lambda	*	34		yes			hyperviscosity
19	SMM	59	2011	148	IgG lambda		IgG lambda	15%	13		no			
20	SMM + Amyloidosis	53.4	2012	76	no	no	kappa	11%		62	yes			Cardiac, liver and renal amyloidosis
21	Plasmacytoma	61.7	2004	164	no	no	IgG lambda	0%	4		no			recurrent costal and vertebral plasmacytomas
22	Plasmacytoma	56.2	2013	253	no	IgG lambda	IgG lambda	0%	13		no			recurrent tibial, digestive and cerebral plasmacytomas

PCN: plasma-cell neoplasia. MM: Multiple Myeloma. SMM: Smoldering Myeloma. LCDD: Light-Chain Deposition Disease. M-Spike: Monoclonal Spike.

* = unknown.

#### Infections

**Fig 2:**
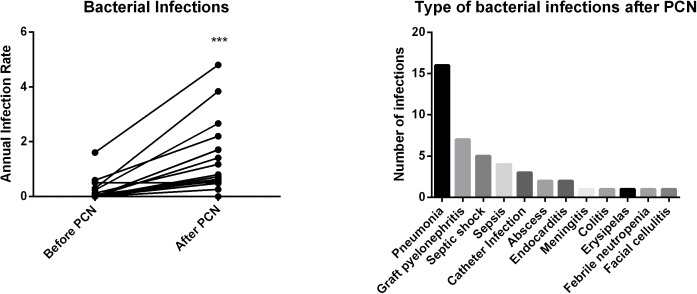
Bacterial infections before and after the diagnosis of plasma-cell neoplasia after kidney transplantation. **A:** The annual rate of bacterial infections was compared for each patient before and after the diagnosis of PCN during the kidney transplantation period. (*** = p<0.001). **B:** Type (x axis) and number (y axis) of bacterial infections during the follow-up of PCN. PCN: plasma cell neoplasia.

There was a particular risk of infection in the first year following the diagnosis of PCN with 19 bacterial infections in 12/22 patients, four viral infections, and two opportunistic infections (one pulmonary aspergillosis and one candidemia). During the follow-up, the most frequent bacterial infections were pneumonia and graft pyelonephritis. The annual bacterial infection rate was significantly higher after the diagnosis of PCN, rising from 0.16 (±0.37) to 1.09 (±1.30) (p = 0.0005). Most of the bacterial and fungal infections occurred during treatments of PCNs (36/46, 78%), including 4 of them directly related to autologous stem cell transplant (ASCT). Data regarding bacterial infections are presented in **Fig 2**.

Conversely, the annual viral infection rate was not significantly different before and after the occurrence of PCN (0.09 versus 0.11, p = 0.83). No BK virus associated-nephropathy was observed. EBV receiver serology was positive in 16/16 patients and donor serology was positive in 10/13 patients. Only three patients had EBV replication in blood during the follow-up, including the two patients with plasmacytomas. In situ hybridization using EBV-encoded RNA (EBER) was tested on the tumoral proliferation (two plasmacytomas and one bone marrow biopsy) in these three patients, and was only positive in the patient with multiple plasmacytomas localized to the gut, sternum and brain.

#### Impact on allograft rejections

Eight patients had an acute rejection before the diagnosis of PCN versus none after (36% versus 0%, p = 0.004). Chronic graft dysfunction occurred in four patients, including three biopsy-proven chronic allograft rejections, and was responsible for graft loss in two patients.

#### Immunosuppressive treatment after PCN diagnosis

Except in one patient who returned to hemodialysis, maintenance therapy was reduced in 11 patients (50%) because of severe infections (7/11) or chemotherapy (4/11). The choice to reduce the maintenance therapy in case of severe infections was always taken during or after severe bacterial, viral or fungal infections, and not preventively. The reduction of maintenance therapy alone was never chosen as an option to treat PCNs. The therapeutic adaptations were highly variable. During the first year after diagnosis, three patients had an overall decrease in their immunosuppressive doses, calcineurin inhibitors were replaced by sirolimus in four patients, and mycophenolate mofetil was stopped in one patient. During follow-up, calcineurin inhibitors were stopped in one patient, and two patients had all immunosuppressive treatments discontinued because of severe infections.

#### Treatment(s) for PCN

The 16 patients with symptomatic MM (12 initial MM and four SMM evolving to MM) were treated. One patient received only dexamethasone, and severe infection led rapidly to death before further treatment could be applied. Three patients diagnosed before 1998 were treated with melphalan-prednisone; two of the patients died shortly thereafter. Bortezomib regimen treatments were used as first-line in 10 patients. Five had no relapse at the end of follow-up, and were all alive at the end of the study, with a median follow-up of 15 months (range 11–41). Relapsing patients all died after other second-, third- or fourth-line chemotherapies, with a median survival of 32 months (range 14–98).

Among 13 patients under the age of 65 eligible for ASCT, four received this treatment after first-line chemotherapy, and one after two lines of chemotherapy. Four patients died before they received a transplant. ASCT was probably contraindicated in one patient because of graft failure. Three patients did not receive ASCT for unknown reasons. Treatments, responses and outcomes are presented in **Table 3.**

**Table 3:** Treatments, responses and outcomes of the plasma-cell neoplasias.

Patient	Date of diagnosis	Initial/Final diagnosis	1st treatment	Response to the 1st treatment	Progression free survival after the first treatment (in months)	2nd	Response to the 2nd treatment	3rd	Response to the 3rd treatment	4th	Response to the 4th treatment	Outcome	Cause of death	Follow up from diagnosis, in months	Follow up when symptomatic MM (if different) in months
1	1991	MM	MP	PR	2							Dead	Aspergillosis during treatment	3	
2	1997	MM	MP + Radiotherapy	PR	49							Dead	Stroke	49	
3	2005	MM	TD	SD	91	RD	PR					Alive		91	
4	2006	MM	BD + cementoplasty then ASCT	VGPR	52	TD	Progressive disease					Dead	Progressive disease	56	
5	2006	MM	CD then ASCT	SD	14	BD	unrated					Dead	Septic shock during treatment	20	
6	2006	MM	Dexamethasone	unrated	x							Dead	Pneumonia before initiation of treatment	2	
7	2010	MM	BMP	Progressive disease	4	RD (continuous treatment)	VGPR					Dead	Septic shock during treatment	17	
8	2010	MM	BCD	SD	14	Thal-Dex etoposide	SD	Benda-M	SD			Dead	Progressive disease	14	
9	2012	MM	BCD	VGPR	13							Alive		13	
10	2013	MM	BCD then ASCT	CR	15							Alive		15	
11	2013	MM + plasmacytoma	BTD then ASCT	VGPR	19							Alive		19	
12	2013	MM	BCD	PR	11							Alive		11	
13	1996	SMM / MM	MP	unrated	2							Dead	Pneumonia during treatment	50	2
14	2004	SMM / MM	BD	VGPR	12	RD	VGPR	CD	Progressive disease			Dead	Sepsis during treatment and progressive disease	98	26
15	2009	SMM / MM	BMP	PR	9	VCMP	PR	BD	PR	RD	SD	Dead	Progressive disease	32	26
16	2009	SMM + LCDD	BD	VGPR	12	BCD	PR	RD (continuous treatment)	SD			Dead	Sepsis during treatment and stable disease	48	
17	2010	SMM / MM	BD	VGPR	23							Alive		41	23
18	2011	SMM / SMM + hyperviscosity	BD	PR	14							Alive		20	14
19	2011	SMM			x							Alive		21	
20	2012	SMM + Amyloidosis	BCD	unrated	8							Dead	heart failure secondary to amyloïdosis	8	
21	2004	Isolated / Multiple plasmacytomas	Corporectomy Radiotherapy	CR	13	Corporectomy Radiotherapy	CR	VD	Progressive disease	RD then ASCT	CR	Alive		106	
22	2013	Isolated Plasmacytomas	BD (one cycle) Radiotherapy	CR	5	Radiotherapy	CR	BMC	SD	ICE + Radiotherapy	Progressive disease	Dead	Progressive disease	19	

PCN: plasma-cell neoplasia. MM: multiple myeloma. SMM: smoldering myeloma. MP: melphalan prednisone. TD: thalidomide dexamethasone. VD: velcade dexamethasone. ASCT: autologous stem cell transplantation. VMP: velcade melphalan prednisone. VCD: velcade cyclophosphamide dexamethasone. VTD: velcade thalidomide dexamethasone. RD: revlimib dexamethasone. CD: cyclophosphamide dexamethasone. Benda-M: bendamustine medrol. VCMP: vincristine cyclophosphamide melphalan prednisone. VMC: velcade melphalan cytarabin. ICE: ifosfamide cytarabin etoposide. PR: partial response. SD: stable disease. VGPR: very good partial response. CR: complete response.

#### Outcome of patients and kidney grafts

Thirteen patients (59%) died during follow-up. Seven patients died because of severe bacterial or fungal infection, occurring during treatments for multiple myeloma in six of them, and before initiation of treatment in one patient. Four patients died because of disease progression after two or more regimen treatments. The patient with AL amyloidosis died from cardiac failure. The last death (from a stroke) was not related to PCN.

**Fig 3:**
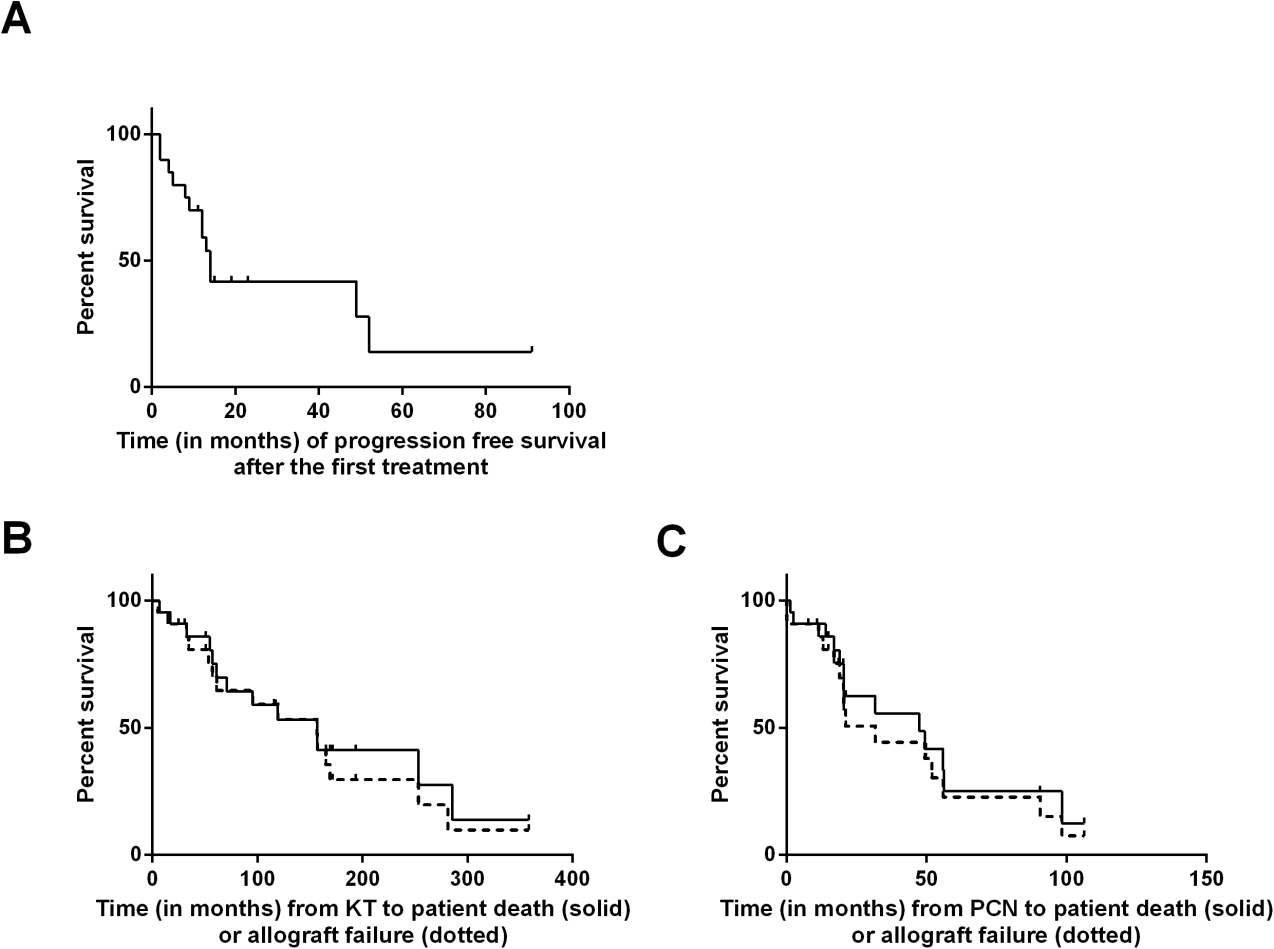
Graft and patient survival. **A:** Progression free survival (in months) after the first treatment for PCN. Median progression free survival (20/22 patients) was 14 months. **B:** Survival (in months) from kidney transplantation (KT) of patients (solid) and grafts (dotted). Median graft survival after KT was 156.9 and median patient survival after KT was 156.9 months. **C:** Survival (in months) from the time of diagnosis of PCN of patients (solid) and grafts (dotted). Median graft and patient survival after diagnosis of PCN was 31.7 months and 49.4 months, respectively. PCN: plasma cell neoplasia.

Serum creatinine was higher at the time of diagnosis of PCN than before, increasing from 135.7 (±71.6) to 195.9 (±123.7) μmol/l (p = 0.008). This impact on graft function was explained by the specific PCN-related kidney graft failure already described. Of the 15 grafts lost (68% patients), 12 (80%) were caused by PCN. Nine graft losses were linked to the patient’s death, including eight deaths directly attributable to PCN. Four graft losses were associated with specific kidney graft failure: two because of LCDD, respectively 4.4 and 1.0 years after diagnosis, and two because of cast nephropathy immediately after diagnosis. Only three graft losses were unrelated to PCN, two because of chronic graft dysfunction, respectively 9.6 and 13.7 years after renal transplantation, and one from death due to a stroke. Median progression free survival after the first treatment for PCN (20/22 patients) was 14 months [2–91]. Median graft and patient survival after KT were equal at 156.9 months. Median graft survival after the diagnosis of PCN was 31.7 months and median patient survival after the diagnosis of PCN was 49.5 months **(Fig 3)**. Removing the three patients diagnosed before 2002 treated with Melphalan regimens gave similar results: median graft and patient survival after KT were equal at 157.3 months, median graft survival after the diagnosis of PCN was 21,1 months, and median patient survival after the diagnosis of PCN was 47,4 months (**S1 Fig**).

**Fig 4:**
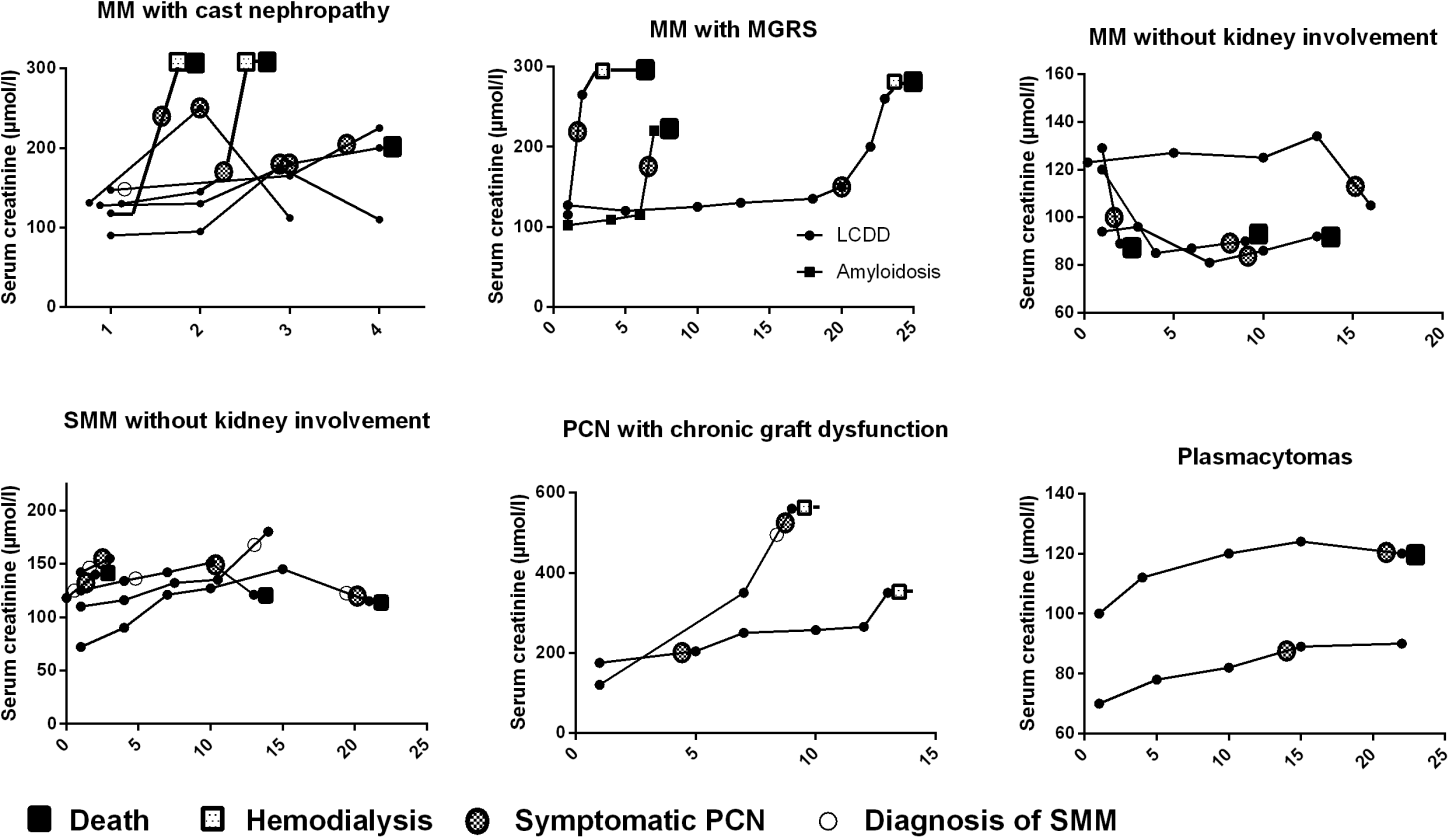
Evolution of serum creatinine after kidney transplantation, combined with the occurrence of asymptomatic or symptomatic PCN, hemodialysis and death for each patient. Evolution of serum creatinine (μmol/l) over time (in years) after kidney transplantation, for each patient. Patients were classified in six groups, and approximate time of diagnosis of SMM (smoldering multiple myeloma), symptomatic PCN (plasma-cell neoplasia), hemodialysis and death were added. MM: Multiple Myeloma. MGRS: Monoclonal Gammapathy of Renal Significance. LCDD: Light Chain Deposition Disease.

**Fig 4**shows the evolution of serum creatinine after KT, combined with the occurrence of asymptomatic or symptomatic PCN, hemodialysis and death for each patient.

## Discussion

PCNs in KT are rare. The prevalence calculated in our study was analogous to the one reported in the United States [8], around 1/1000. In the present study, MM occurred at any time after KT, and the incidence seems to be steady over time post-transplantation. Plasmacytoma develops later in the post-transplantation period. Although rare, PCN after KT occurs in younger patients compared to the general population, in which the median age of PCN at diagnosis is over 70 [5].This may be mostly due to the fact that KT patients belong to a selected population, i.e., in France the median age is 55 years. Older patients, who are at risk of developing PCN, are less often transplanted. Engels et al.[8] made the same observation in their epidemiologic study in the United States regarding PCN after solid organ transplantation.

In the general population, MGUS is a premalignant plasma cell proliferative disorder with a constant risk of progression to MM. In KT, monoclonal gammopathy is 10 times more frequent than in the general population, but is not associated with a high occurrence of PCN [33–36]. Most of these MGUS are transient, and probably reflect the temporary loss of control of the B-cell lymphoid population secondary to immunosuppressive therapy [33,34,37]. There is probably no risk of these particular transient MGUS, which differ from classic MGUS, evolving to MM.

The fact that classical MGUS was often present before KT in our study does not argue in favor of PCN being classified as PTLD, but raises the question of a pre-transplant lymphoproliferative disorder for some of the cases. Onset kinetics of PCN was not influenced by presence of MGUS before KT; however, presence of light chain was not systematically screened before KT. Although it is not given in the KDIGO recommendations, such screening could be more systematic, before and after KT. At the moment, development of a clonal plasma cell may occur either before or after KT, and it is not clear that KT influences the onset of MM.

It should be noted that two patients with plasmacytomas had no MGUS before KT, developed their disease more than 10 years after KT, and thus had definitive PTLD.

A virus or immunosuppression may favor the onset of PCN after KT. The two patients with plasmacytomas had EBV replication in blood, with evidence of EBV replication in situ in one of them, and published data strongly suggest a link between plasmacytoma and EBV infection [8,14–16,18,19,21,24,38,39]. The role of EBV in onset of myeloma is less clear, and this also suggests that plasmacytoma and myeloma may have different courses of pathophysiological development.

Immunosuppressive therapy could also accelerate the development of the hematologic disease. In post-transplant lymphomas, immunosuppressive depletive treatments lead to the loss of control of the B-cell population and clearly favor the onset of hemopathy [40]. Conversely, immunosuppressive therapy may have a beneficial effect. For example, the inhibition of the mammalian target of rapaymycin/Akt pathway has been associated with an anti-tumor effect in MM cells treated with pomalidomide [41]. Mycophenolate mofetil has an anti-proliferative effect on MM in vitro [42], and cyclosporine may sensitize MMs tumoral cells to chemotherapy in vitro [43]. Nevertheless, these results are not sufficient to form a conclusion about the effects of immunosuppressive therapy on PCN in vivo in KT. Furthermore, cyclosporine had no additional effect when it was combined with vincristine doxorubicin and dexamethasone in a phase II/III clinical trial in patients with advanced refractory MM [44].

MM occurring after KT shares the same clinical characteristics as MM in the general population. SMMs often become symptomatic MMs. Bone lesions, anemia, hypercalcemia and kidney failure are the common manifestations in renal transplant patients. Progression to plasma cell leukemia was even diagnosed in one patient. The kidney graft was a potential target of the hematologic disease as it would be for native kidneys, and presented the same histological lesions, such as cast nephropathy, LCDD or AL amyloidosis.

The patients in the present study showed a substantial increase in the number of bacterial infections after the diagnosis of PCN, especially during treatments. This suggests that patients were highly immunocompromised. On the other hand, acute rejections prior to diagnosis of PCN were frequent. This suggests that immune stimulation plays a role in the development of disease, or enhances the potentially harmful nature of the immunosuppressive therapy which was probably increased after the rejection episode. Conversely, no acute rejection occurred after the diagnosis of PCN, despite a decrease of immunosuppressive therapy in half of patients. The immunosuppressive effects of PCN certainly decrease the allogeneic response of the transplanted kidney as it reduces the response to bacterial attacks. All these facts suggest that immunosuppressive treatments should be drastically and preventively reduced in KT patients with symptomatic PCN, and even be discontinued in the case of severe infectious complications. The risk of rejection is indeed much lower than the risk of infection and most of these patients are treated with high doses of corticosteroids and bortezomib, also used in the treatment of transplant rejection.

Contrary to the recent study of seven patients by Safadi et al. [29], the present findings suggest that KT patients die early from PCN. Median progression free survival was low at 14 months, compared to 21 months in the classical association of bortezomib plus dexamethasone for the frontline treatment of multiple myeloma [45]. Median survival is almost twice as low as the current median survival in the general population [30]. This increased mortality is not explained by the long period of inclusions, because it remains when the three patients treated with melphalan are removed from the analysis. Severe bacterial or fungal infections occurring during treatments for PCNs were the principal cause of death. The conjunction of maintenance therapy for kidney graft and treatments for PCN may favor the risk of lethal infections in these patients. The control of PCN by auto-immunity may also have been compromised by the immunosuppressive therapy. In our study, PCN also had a huge impact on graft outcomes, at the time of diagnosis because of specific kidney failure, and later as a cause of the majority of graft losses. Survival of graft and patients was indeed very similar, closely dependent on the hematologic disease.

The treatments were heterogeneous, and reflected the rapid evolution of chemotherapies during the last twenty years. From 2004, the principal first-line treatment was a combination of chemotherapy using bortezomib and dexamethasone, with or without another agent (primarily, cyclophosphamide or thalidomide). MM occurring after KT should be treated with the same rigor as in the general population, according to the most recent guidelines. In particular, ASCT was not systematically proposed for all eligible patients; this treatment should be more widely applied.

There are several limitations to this study. It was retrospective in nature, and because of the rarity of the disease patients were included over a long period of time. Because hematological disease differed depending on whether it was MM, SMM or plasmacytoma, patients were heterogeneous. Immunosuppressive treatments and chemotherapy were equally diverse, and it was not possible to precisely analyze their potential multiple effects on the evolution of PCN. Further prospective studies are needed to refine the epidemiology and outcome of these patients, and to explore ways of improving the prognosis. Despite these limitations, the present study is to our knowledge the largest case series of descriptive PCN after KT, and already provides useful findings.

## Supporting information

S1 Fig Graft and patient survival after 2002. The three patients diagnosed before 2002 treated with Melphalan regimen were removed from the analysis. **A:** Survival (in months) from kidney transplantation (KT) of patients (solid) and grafts (dotted). Median graft survival after KT was 157.3 and median patient survival after KT was 157.3 months. **B:** Survival (in months) from the time of diagnosis of PCN of patients (solid) and grafts (dotted). Median graft and patient survival after diagnosis of PCN was 21.1 months and 47.4 months, respectively. PCN: plasma cell neoplasia.
(TIF)
